# ART integration in oral health care systems in Latin American countries as perceived by directors of oral health

**DOI:** 10.1590/S1678-77572009000700018

**Published:** 2009

**Authors:** Oswaldo RUIZ, Jo E. FRENCKEN

**Affiliations:** 1 DDS, Senior Lecturer, Department of Postgraduate Studies, Central University of Ecuador, Quito, Ecuador.; 2 DDS, MSc, PhD, Associate Professor, Department of Global Oral Health, Radboud University Nijmegen Medical Centre, College of Dental Sciences, Nijmegen, the Netherlands.

**Keywords:** Atraumatic Restorative Treatment (ART), Science transfer, Latin America, Health care systems, Health policy

## Abstract

The aim of this study was to carry out a situation analysis of: a) prevalence of ART training courses; b) integration of ART into the oral healthcare systems and; c) strengths and weaknesses of ART integration, in Latin American countries. Materials and Methods: A structured questionnaire, consisting of 18 questions, was emailed to directors of national or regional oral health departments of all Latin American countries and the USA. For two countries that had not responded after 4 weeks, the questionnaire was sent to the Dean of each local Dental School. The questions were related to ART training courses, integration of ART in the dental curriculum and the oral healthcare system, barriers to ART implementation in the public health system and recommendations for ART implementation in the services. Factor analysis was used to construct one factor in the barrier-related question. Means and percentages were calculated. Results: The response rate, covering 55% of all Latin American countries, was 76%. An ART training course had been given in all Latin American countries that responded, with more than 2 having been conducted in 64.7% of the respondent countries. ART was implemented in public oral health services in 94.7 % of the countries, according to the respondents. In 15.8% of the countries, ART was applied throughout the country and in 68.4%, in some areas or regions of a country. ART had been used for more, or less, than three years in 42.1% and 47.4% of the countries, respectively. evaluation and monitoring activities to determine the effectiveness of ART restorations and ART sealants had been carried out in 42.1% of the countries, while evaluation training courses had taken place in only 3 countries (15.8%). Respondents perceived the “increase in the number of treated patients” as the major benefit of ART implementation in public oral health services. The major perceived barrier factors to ART implementation were “operator opinion” and “high patient load”, followed by “lack in supplies of materials and instruments and operators” and “lack of ART training”. Respondents recommended that the number of ART courses should be increased. Conclusions: The introduction of ART into the public oral health systems in Latin American countries has taken place but is still in its infancy. More ART training courses need to be organized if the approach is to be adopted in oral health service systems in these countries.

## INTRODUCTION

In many developing countries, access to and provision of oral health care is limited^12^. Characteristically, the levels of untreated cavitated lesions are high. As the option of saving a painful tooth by placing a restoration is often not considered, because of a lack of functional dental equipment and materials, and because of the acceptance by patients that toothache can be alleviated only through extraction of a badly decayed tooth, toothache is usually treated by extraction^5^. This situation has arisen as a result of the unconditioned acceptance by governments and professionals in low- and middle-income countries of inappropriate oral healthcare models. These are based on rotary-driven equipment and, although this type of health care has a place in developing countries, their use is more suited to high-income countries having the required infra-structure. In order to improve the situation in developing countries, their authorities need to identify oral care models that suit their health conditions, means and healthcare infra-structure.

One such approach, considered by World Health Organization (WHO) as appropriate for use in low- and middle income countries, is the Atraumatic Restorative Treatment (ART) approach. It was officially adopted by the World Health Organization in 1994 as a technique that could contribute to the control of dental caries, as part of primary oral health programs in developing countries^15^. The restorative component of the ART approach is based on using only hand instruments to eliminate soft, demineralized carious tooth tissues. In the majority of cases, the cleaned cavity is restored with a high-viscosity glass-ionomer^14^. As it does not require electricity or expensive dental equipment, ART offers a pragmatic solution for the problems related to the prevention of carious lesion development and progression and the restoration of untreated cavitated carious lesions^3^^,^^8^.

Makoni, et al.^6^ (1997) showed that ART could be applied in 84% of dentine cavities in an adolescent population with a caries prevalence of 41% and a mean DMFT score of 1.1. The longevity of single-surface ART restorations in primary and permanent tooth has been reported to be good^8^. Mickenautsch, Yengopal and Banerjee^8^ (2009) found no difference in survival results, after six years, between single-surface ART restorations and comparable amalgam restorations in the permanent dentition.

The preventive component of ART, that is the sealing of caries-prone pits and fissures with a high-viscosity glass-ionomer, also showed good results, with an annual dentine lesion development of only 1% during the first three years of placement^14^.

The cost-effectiveness of amalgam and ART restorations using high-viscosity glass-ionomer was studied in three Latin American countries: Panama, Ecuador and Uruguay. The results showed that single-surface ART restorations in permanent teeth were more cost-effective than comparable amalgam restorations after two years. On the basis of this finding, Pan-American Health Organization (PAHO) recommended the introduction of ART into oral health policies in Latin American countries^10^.

The evidence demonstrates that the ART approach produces quality sealants and quality restorations in single-surfaces both primary and permanent teeth. Thus the time has come to extend the structured introduction of ART into the national oral health policies of more low- and middle-income countries than those from which reports regarding its efficacy have been received: South Africa^7^, Tanzania^5^ and Mexico^4^.

### Science transfer

One of the most important, but at the same time very difficult, aspects of research is the transfer of results of studies into daily medical/dental practice. The main difficulty is to get practitioners to accept, adopt and apply newly obtained evidence-based results. Educating dental students for life-long learning in dental schools worldwide is only a recent development. Personal experience shows that many dental schools have not adopted the problem-based learning concept. These continue to use the conventional teacher-student one-directional education system. It is not surprising that professionals educated in this way have great difficulties in accepting new developments in medicine and dentistry. Rindal, et al.^13^ (2008) noted that clinical inertia, resistance to accepting newly developed treatments in medicine/dentistry, is a useful paradigm for explaining delays in the incorporation of new knowledge in clinical practice. Introducing the ART approach into oral healthcare systems in a sustainable manner under such prevailing conditions would be difficult.

### ART introduction in Latin America

ART has been introduced into oral healthcare systems in Latin American countries. In Peru a basic comprehensive oral health project that included ART was implemented in primary schools in a large number of deprived communities ten years ago^9^. ART is now integrated within the national oral health policy of Peru. In Chile the Ministry of Health has developed an oral health program called: “An Integral Clinical Oral Health Guide for 6 year-old children”^1^. It attempts to manage dental caries development and progression through sealing pits and fissures, the use of additional caries control measures and ART restoration of tooth cavities. As early as 1998, an ART course was organized in Mexico City. This formed the basis for the development of an oral health program for underserved Mexican provinces, covering 25 million people^4^.

PAHO has recommended the adoption of ART in oral health services in Latin American countries but no evaluation report to this effect were available in the literature. Therefore the decision was made to carry out a preliminary situation analysis of the: a) prevalence of ART training courses; b) integration of ART into the oral healthcare system and; c) strengths and weaknesses of the ART integration in Latin America.

## MATERIALS AND METHODS

### Questionnaire

**Figure 1:**
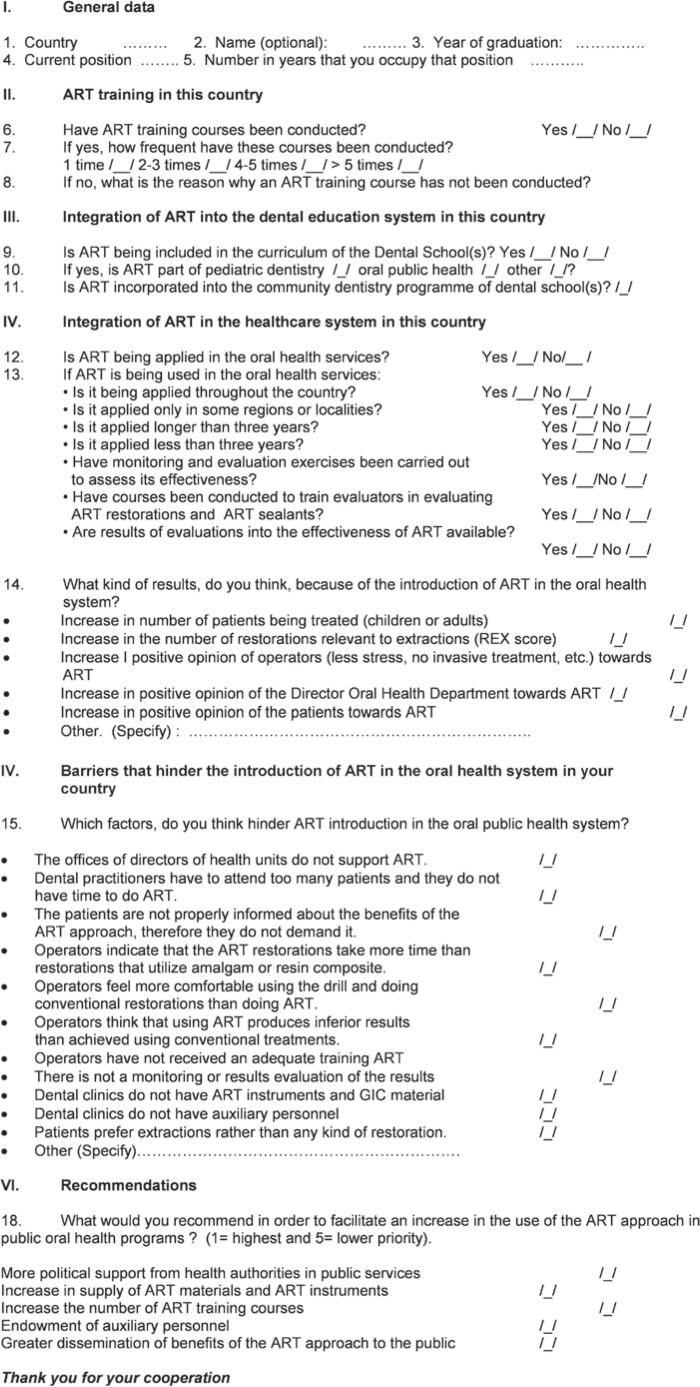
Questionnaire assessing ART integration into oral health care systems in Latin American countries

A structured questionnaire, consisting of 18 questions, was sent through the internet to directors of national or regional oral health departments of all Latin American countries and the USA between April and July 2009 (Figure 1). A reminder was sent after four weeks. For the two countries that had not responded, the questionnaire was also sent to deans of dental schools.

### Construction of variables

Factor analysis was performed for four items of the barrier question (Q15) to construct one factor, “operator opinion”, which had a Cronbach's alpha of 0.62. All other barrier factors were single item statements.

### Statistical analysis

Microsoft excel software was used for entering data onto the computer and checking for accuracy. The data were then transferred into an SAS program for analysis by a statistician. A question that was not answered was considered as “not being in agreement”. Mean scores and percentages were calculated.

## RESULTS

### Disposition of subjects

From the total of 25 questionnaires sent, 19 were returned from 10 Latin American countries and 1 from the USA, covering 55% of all Latin American countries (Table 1). The respondents were directors of national (42.1%) and regional (47.3%) oral health departments, and university lecturers (10.5%). Most of the respondents (52.9%) had graduated before 1990 and 76.5% had held the position of director of oral health for less than 3 years. Only 4 directors (23.5 %) had held that position for 9 or 10 years.

**Table 1:** Frequency distribution (%) of participating countries

Country	Frequency	Percent
Chile	6	31.6
Ecuador	3	15.8
El Salvador	2	10.5
Honduras	1	5.3
Mexico	1	5.3
Nicaragua	1	5.3
Panama	1	5.3
Paraguay	1	5.3
Peru	1	5.3
Uruguay	1	5.3
USA	1	5.3

### ART education through training courses and dental curricula

An ART training course had been conducted in every country, with the majority of countries (64.7%) having received 2-3, and 23.5% having received 4 or more ART training courses. Whether the ART approach was part of the dental school curriculum, was answered affirmatively by 73.7% of the respondents. ART training was included in pediatric dentistry (21.1%), public oral health (10.5%) and other dental courses (15.8%). Inclusion of ART in the community dentistry program of a dental school was affirmed by 31.6% of the respondents.

### Implementation of ART in oral care systems

Almost all recipients (94.7%) responded that ART had been implemented in the nation's public oral health services; 15.8% stated that ART was used throughout their country, while 68.4% commented that it was used in only some areas or regions of their country. Only 2 stated that ART was used in private practices. With regard to the length of period that ART had been in used in these countries, 42.1% of the respondents indicated that it had been applied for more than three years, and 47.4% indicated that it had been applied for less than three years.

Regarding the evaluation and monitoring activities for determining the quality of ART restorations and sealants; 42.1% of respondents answered affirmatively. However, courses for training evaluators in assessing the quality of ART sealants and restorations had been held in only 3 countries (15.8%).

### Strength and weakness of ART implementation

Table 2 presents the responses to the question regarding the perceived results of the introduction of ART into the public health services. The most important result noted by the respondents was the “increase in number of patients treated”.

**Table 2:** Mean and standard deviation (SD) of perceived benefits from ART implementation in public oral health services in Latin American countries by directors of oral health

Perceived benefits	Mean	SD
Increase in number of patients treated	0.63	0.49
Increase in positive opinion on ART by operators	0.53	0.51
Increase in positive opinion on ART by patients	0.47	0.51
Increase in positive opinion on ART by Director of Oral Health	0.21	0.49

With respect to identifying barrier factors inhibiting implementation of ART; “operator opinion” and “high patient load”, followed by “lack in supplies of materials and instruments” and “operators lack of ART training” were perceived by respondents in these countries to be the most important, as shown in Table 3.

**Table 3:** Mean and standard deviation (SD) for perceived barrier factors to ART implementation in public oral health services by directors of oral health in a number of Latin American countries

Barrier factors	Mean	SD
Operator opinion	0.43	0.26
Patient load	0.42	0,51
Lack in supplies of materials and instruments	0.37	0.50
Insufficient skills to carry out ART	0.32	0.48
Absence of chair side assistant	0.26	0.45
No support from management	0.21	0.49

Table 4 shows the recommendations, in descending order of importance that would facilitate the implementation of ART in the public oral health services of these countries. Organizing ART training courses in the participating countries was considered to be the most important recommendation.

**Table 4:** Recommendations (mean score and standard deviation; SD) by directors of oral health to facilitate further implementation of ART in public oral health services in Latin American countries

Recommendations	Mean	SD
To organize ART training courses	2.1	1.3
To provide more political support by health authorities	2.4	1.2
To ensure availability of materials and instruments	2.7	1.0
To have auxiliary personnel available	3.6	1.4
To disseminate the benefits of ART to the profession and public	3.7	1.5

1 is the highest and 5 is the lowest priority score

## DISCUSSION

The purpose of the present study was to investigate aspects of the integration of the ART approach into oral health care in Latin America. The response rate was 76%, which implies that some caution should be taken when interpreting the results. Furthermore, a questionnaire like the present one, which relied on information available at the offices of directors of the departments of oral health of the ministries of health, may contain a certain level of bias. The value of the supplied information is dependent upon the organizational structure of each department, which might (or might not) have made available all the requested information about the present situation in its country.

Although the findings of the present study should be considered with some caution, the fact that ART courses have been conducted in all participating countries shows that the directors are aware of ART and that they and others in authority intend to introduce ART into their national healthcare systems. This finding is supported by the knowledge that ART is part of the national oral health programs of countries like Brazil, Chile, Ecuador, Peru, Mexico and Uruguay. Further evidence of ART integration comes from the finding that ART has been included in the *curricula* of dental schools in a number of countries; such as Argentina, Bolivia, Brazil, Ecuador, Mexico, Peru and Venezuela. The inclusion of ART in the dental *curricula*, though not on a massive scale, clearly indicates that the authorities intend to make the ART approach available for use by practitioners in public and private practice. Research monitoring the effects of ART introduction and assessing the quality of ART restorations and ART sealants has been conducted in some Latin American countries, though not on a wide scale. The present study found that ART evaluation courses had been given on relatively few occasions. Therefore, increased implementation of aspects of research methodology appears to be needed in these countries, aimed at monitoring ART integration into their oral health service systems and scientifically reporting the findings. Mexico serves as an example of this suggestion^4^. On the basis of the above findings, the conclusion was reached that implementation of the ART approach in Latin American countries is still in its infancy stage.

Implementation of innovations and new developments has generally been met with resistance and ART has not been immune to this. If the probability of a wider acceptance of ART in oral care is to be increased, reasons for possible resistance need to be elicited. The barrier factors reported most frequently in the present study were: “operator opinion” and “patient load”, followed by “absence of sufficient practical skills” to enable dental practitioners to produce quality ART sealants and ART restorations and the “absence of sufficient ART instruments and restorative materials”. The “operator opinion” and “absence of sufficient practical skills” barrier factors can be overcome by increasing the number of ART courses given by experienced ART trainers in the countries. Such training could be included in the national oral health programs. Spanish and Portuguese ART manuals are available and professional ART teachers can be trained through adoption of the “Train the Teacher” concept, as done in Mexico^4^, with assistance of the Department of Global Oral Health in Nijmegen, the Netherlands.

The absence of the relatively few ART instruments (only 5) and glass-ionomer material have been reported as factors negatively affecting the introduction of ART into the oral health services of South Africa^7^ and Tanzania^5^. Coordinated efforts between representatives of the ministries of health, national dental associations and industry could determine ways of ensuring the availability of quality ART instruments for service providers in Latin American countries. A cautionary point should be noted here. Over the last decades, many different brands of glass-ionomer restorative materials have been marketed all over the world. On the basis of the finding that the use of medium-viscosity glass-ionomers with ART had produced ART restorations in single-surfaces that were inferior to those produced when using high-viscosity glass-ionomers^14^, dental practitioners and authorities in charge of purchasing glass-ionomer material should opt for quality and field-tested high-viscosity glass-ionomer restorative material, instead of opting for the cheapest glass-ionomer, which may be far less effective. Using field-tested high-viscosity glass-ionomers in the hands of trained dental practitioners will produce long-lasting ART sealants and ART restorations that will benefit the health of the general public. The production of quality ART restorations has been demonstrated in the study carried out in Ecuador, Panama and Uruguay^10^. The 2-year survival rate of ART high-viscosity glass-ionomer restorations was very high and was equal to that of comparable amalgam restorations. In summary: appropriate training in ART at the under- and postgraduate levels and adequate provision of the tools and quality glass-ionomer would be key factors affecting the adoption and proper implementation of ART in oral health services in Latin American countries.

Because of the high level of dental caries in the youth in many Latin American countries^11^, and the insufficient preventive and restorative care available to communities there, health authorities in Latin American countries need to work towards improving the oral health services. They need to make proper use of the existing resources in each health unit; perhaps training dental auxiliaries instead of dentists. This would enable them to address the high patient load barrier factor. They would also need to ensure the availability of adequate materials, instruments and dental equipment. Without these guidelines and specific targets, and without a monitoring system managed by competent suitably trained people, dental practitioners may tend to ignore the need to introduce new and evidence-based health methods into their daily practice and consequently, provide very little information to patients about the benefits.

The World Health Organization (WHO) strongly recommends the implementation of the Basic Package of Oral Care (BPOC) adjusted to the actual conditions of each community^2^. ART, being a part of this package, has been recommended for use in Latin American countries by the Pan American Health Organization. Countries that wish to implement the BPOC or only ART may first have to overcome the barrier factors identified in this study, before starting to introduce BPOC and/or ART into their oral healthcare systems.

ART training courses have been conducted in all participating Latin American countries. ART has been introduced, to varying degrees, into public oral health systems of almost all the participating countries and the main barrier factors for ART implementation are operator opinion, high patient load, insufficient skills for implementing the ART approach, and insufficient availability of restorative materials and ART instruments. The introduction of ART in Latin American countries appears to be still in its infant stage. The highest recommended priority to consider regarding further introduction of the ART approach is the organization of ART training courses.

### Recommendations

In order to facilitate the integration of ART into the national oral healthcare systems of Latin America, the relevant authorities should:

- organize “Training the Trainer” courses in ART, in addition to regular full-level ART courses in countries that have already organized such courses;
- support course participants by ensuring the availability of sufficient ART instruments and a constant supply of quality high-viscosity glass-ionomer restorative material;
- ensure the installation of a system for monitoring treatments provided in public oral health services, which includes assessment of the quality of ART sealants and ART restorations, as well as for caries control measures;
- organize meetings for updating dental practitioners about monitored results;
- promote cooperation of the universities with the ministries of health in developing the ART oral health project.
